# Dyslexic doctors, an observation on current United Kingdom practice

**DOI:** 10.15694/mep.2017.000060

**Published:** 2017-03-23

**Authors:** Michael Kinsella, Mohammed Abdul Waduud, John Biddlestone

**Affiliations:** 1 NHS - Greater Glasgow and Clyde; 2 NHS - The Leeds Teaching Hospital Trust

**Keywords:** Dyslexia

## Abstract

This article was migrated. The article was marked as recommended.

Dyslexic doctors, an observation on current United Kingdom practice.

Issue:

Dyslexia is a common learning difficulty with an estimated prevalence of ten percent within the general population and two percent among junior doctors training in the United Kingdom. Despite dyslexia being common, there are still many challenges sufferers face in modern medical practice.

Evidence:

Multiple case studies have found there to be barriers that dyslexic doctors face throughout their training. Common activities that required reading or writing in time pressured situations in front of an audience can impose an additional pressure for dyslexic doctors. In addition to the difficulties with day to day work, criticism and mockery from other staff members can make suffers of dyslexia feel undermined. From personal experiences, the authors of this article have found barriers are particularly present with regards to sitting post- graduate examinations and getting support in a modern time pressure health service.

Implications:

The discrepancy in the prevalence of learning difficulties between the general population and doctors in training might be due to barriers in training and difficulties when starting work. Addressing challenges will help support current dyslexic doctors and also help support future generations. Rapidly developing technology in health care makes it easier to accommodate doctors with additional needs but the impact of this are yet to be studied. If the barriers are addressed it is likely to support not only doctors with dyslexia diagnosis but all health care professionals.

## Observation Article - Dyslexic doctors, an observation on current United Kingdom practice

Dyslexia affects a significant proportion of the population but there is at present relatively little information about the proportion of medical students and doctors with this condition. The British Dyslexia Association defines dyslexia is a “specific learning difficulty that mainly affects the development of literacy and language related skills.” Dyslexia is common, the prevalence in the UK population is between 5-10%, depending on the definition of dyslexia used (Dyslexia Scotland, 2015) ( Miles, 2004). Dyslexia is common worldwide and there are sufferers in alphabetic languages (such as English and French) as well as non-alphabetical languages, such as Chinese (Siegel, 2014). A recent study from Scotland estimated that 2% of UK medical students suffer from dyslexia ( Newlands, Shrewsbury, & Robson, 2015). This difference in prevalence, with fewer medical students with the condition compared to the general population, could be due to non-disclosure of dyslexia, a failure of a medical school to admit dyslexic medical students or an unwillingness of university applicants with dyslexia to consider a career in medicine. Failure to disclose may be due to a number of factors. In this article, we will try to present some of the reasons for non-disclosure from our personal experiences, and suggest possible improvements in practice.

There is no legal requirement in the UK to disclose dyslexia, but if a person declares they have dyslexia then their employers should ensure they are not treated unfavourably and are offered reasonable adjustments or support. ( “Legislation | British Dyslexia Association”, 2016). In the United Sates of America, there is similar legislation but there is some variation between States ( Youman, & Mather, 2016). Despite legal protection, some doctors choose to not tell their colleagues about their learning difficulty. A recent qualitative study in Scotland found that non-disclosure was due doctors not wanting to be seen as having “help-seeking behaviour”; or dyslexia being used as an excuse for underperformance. Others felt it may have a negative impact on their careers and lead to bullying. Some felt that they should “just get on with it” as they did not want to be treated any differently to their peers ( Newlands, Shrewsbury, & Robson, 2015). Settings such as ward rounds, educational events (i.e. teaching sessions, conference presentations etc.) and telephone conversations, where information is required to be provided and interpreted immediately caused particular anxiety. A surgical ward wound was given as an example where some of the most junior doctors had to write in the case noted during brief patient encounters. In a separate case study, one doctor in a reported “struggling, getting muddled and humiliated” when put on the spot and asked to speak publicly to report an X-Ray on a ward round ( “GMC | Trainee 4”, 2015). He reported mixing up his right and left, which can be a feature of dyslexia, to the amusement of the medical students. Other tasks that dyslexic doctors struggle with are reading and writing handwritten case notes, writing discharge letters and prescribing medication ( Newlands, Shrewsbury, & Robson, 2015). Academic writing and publishing is another key component of medical training and development as it is often awarded marks during job applications which can be difficult for dyslexia doctors can struggle with.

Two authors of this article are dyslexic, we met in medical school as we were some of the few students who had to sit the exam in the medical school library, away from other students. AW was given support at school and formally diagnosed when at university, while MK was diagnosed at age eight. Throughout medical school, both authors received extra support, such as the use of computers or scribes during exams. We both had computers with specific programs to help with course work. There was a member of staff in the study support base that was the point of contact. Other provisions such as lecture notes before the presentation were suggested but did not always happen, but as they were online following this was never followed up on. Following graduation, we had a meeting with the local training committee to arrange extra support on the hospital ward, but it was deemed that little provision was required. The extra-time to write notes and the quiet environment did not happen, due to pressures on service provision and the lack of space. Over the course of two years of rotations on hospital wards, there were no serious incidents but both authors had multiple informal comments from other members of staff, many who knew that we were dyslexic, about poor grammar or getting out dyslexia “sorted”. The biggest change that we have seen in the introduction of computers to the workplace and the integrated online spell checkers and the prescribing has helped in the work management. When entering specialist training both of us discussed our requirements with our Royal Colleges (the organisations responsible for setting examinations and curriculum for hospital specialities). Both colleges had no formal policy about dyslexia in training other than extra support in exams, consistent with education supervisor advice and to be consistent with the Disability Discrimination Act, i.e. to make reasonable adjustments ( “Disability Discrimination Act 2005”, 2017). Both authors have had no problems in training. In contrast for exams, we have found that their issues in proving the provisions. The colleges allow the exam to be taken in various venues in the country but only provide support and extra time in certain venues, which might not be as convenient. As a consequence both of us had to travel to more distant venues, MW had to travel 45 miles extra to sit his exams and MK had to travel 350 miles more. Whilst both authors appreciate the support offered, have provisions in all exam venues would be welcome.

What is clear from case studies and our personal experience is that dyslexia sufferers have multiple coping strategies. From personal experience, the coping strategies we have used are: doing paperwork in quiet environments; delegating tasks in teams to suit our strengths; use of dictaphones and use of friends or family or colleges to proof read articles and presentations. Newlands described the use of teamwork, technology and time management planning to cope with the difficulties dyslexia presents ( Newlands, Shrewsbury, & Robson, 2015). The British Medical Association (BMA) has a dedicated section on their website targeted at medical students with dyslexia ( “BMA - Studying with dyslexia”, 2015). If a medical student is dyslexic the BMA recommends they should request lecture notes in advance when attending events, should record lectures, should request dedicated time for revision and extra-time during exams, should request extended library loans and also have access to a study support tutor. At the medical school that the authors attended these provisions were standard and put in place the start of year one. These provisions were very useful in medical school and have continued to be a benefit in our careers. A case study on General Medical Council website describe how a undergraduate trainee confidence can be improved if a mentor is used to develop a specific focus learning agreement at the start of training ( “GMC | Trainee 4”, 2015). The strategies we have developed could also be useful if they were incorporated into the wider undergraduate education as they might also help non-dyslexia students or those who are not diagnosed or choose not to inform their university or employer. What is clear from the authors’ experience is the development of widespread adoptions of smartphones and computerised notes is that it help with spelling and grammar, but the impact of these changes in dyslexic doctors and medical students experiences is yet to be studied.

In summary, there appears to be a discrepancy between the prevalence of dyslexia in doctors and the wider population. This might be due to non-disclosure or a perception of lack of support available. In this article, we have shown that there is support available and this is likely to improve with new technology. We know that currently, dyslexic doctors have developed coping strategies to overcome barriers. Some support is available but sometimes this involves significant effort and expense and there is a perceived risk of discrimination. In future incorporating proven support techniques to the wider population of both dyslexic and non-dyslexic persons may be beneficial. If more dyslexics declared themselves it would a great role model for the next generation.

## Take Home Messages

• There is a discrepancy between the prevalence of dyslexia in doctors and the wider population.
• Dyslexic doctors suffer from additional pressures and stress due to their disability.
• Support for dyslexic doctors is available, and new technology might be able to help with this.
• Increase support for dyslexic doctors will be beneficial to wider population of both dyslexic and non-dyslexic persons.

## Notes On Contributors

Michael Kinsella: co-author, added personal perspective to article and performed a literature review to provide evidence base for article and prepare article for submission.

Mohammed Abdul Waduud - co-author, added personal perspective to article and came up with original idea of an article. Also helped structure of article and proof read article before submission.

John Biddlestone - provided advice on preparing article for submission and structure of academic writing, help to develop the idea of the article.
