# Supplementary material for: Dyslexic doctors, an observation on current United Kingdom practice
Source: MedEdPublish (2016). 2017 Mar 23;6:60. [Version 1] doi: 10.15694/mep.2017.000060 (PMC10885248; doi:10.15694/mep.2017.000060)
Supplement: Peer review history

# Reviewer report: Reviewer response for version 1

**DOI:** 10.21956/mep.19469.r28717

**Published:** 2020-12-15

**Authors:** Michael Drozd

This review has been migrated. The reviewer awarded 5 stars out of 5 Very interesting topic that is not discussed often. Important insights from the authors highlighting more research is required in this area.

Reviewer Expertise:

NA

No decision status is available

# Reviewer report: Reviewer response for version 1

**DOI:** 10.21956/mep.19469.r28715

**Published:** 2018-06-08

**Authors:** Anthony David.M

**Affiliations:** 1 Soho

This review has been migrated. The reviewer awarded 4 stars out of 5 It is interesting to note that the incidence of dyslexia in doctors is lesser probably due to under reporting. However, it is heartening to note that new technology can help these people. The fact that the authors could go hard and pen this article shows that a way forward is available to dyslexics in the medical profession.

Reviewer Expertise:

NA

No decision status is available

# Reviewer report: Reviewer response for version 1

**DOI:** 10.21956/mep.19469.r28716

**Published:** 2017-03-24

**Authors:** Richard Hays

**Affiliations:** 1 James Cook University

This review has been migrated. The reviewer awarded 4 stars out of 5 I think this paper makes a useful contribution to the debate about the impact of dyslexia on medical education. It is a commentary from people with dyslexia and have found ways of doing well despite having dyslexia. The comments on what worked well in overcoming any potential barriers is constructive. Having worked in both the UK and Australia, I have noticed quite sharp differences in reporting, with dyslexia reported less frequently than in the UK. Is this because the stigma is potentially greater in Australia? I suspect that some who want to avoid the stigma do not report because they manage without adjustments and Does extremely high ability compensate for the measured gap in performance measures? One would think that computerisation, with voice recognition and print-to- audio software should reduce disadvantage. Do those diagnosed earlier manage better and become either less or more confident in reporting and seeking help? Some diagnoses as a result of academic performance issues after commencing a medical program. Several research questons may deserve attention.

Reviewer Expertise:

NA

No decision status is available

# Reviewer report: Reviewer response for version 1

**DOI:** 10.21956/mep.19469.r28718

**Published:** 2017-03-23

**Authors:** Trevor Gibbs

**Affiliations:** 1 AMEE

This review has been migrated. The reviewer awarded 4 stars out of 5 This is the second paper in this edition that deals with dyslexia ( other paper being Paper No 29) , which I feel shows the importance of the subject, and the difficulties that are experience in medical education. Although I do not think that the paper adds much to the thinking of those students and graduates who have dyslexia, I do feel that the content should be read by those involved in curriculum planning.Both papers do describe the difficulties encountered by having dyslexia, although this one is a little more positive in its conclusions, and the opportunities that technology brings.I hope that the answer to the discrepancy between the number of dyslexics found in the general population compared to that found within the healthcare student population can be resolved through research in the future .

Reviewer Expertise:

NA

No decision status is available
